# Re-description of *Euryeidonconsideratum* Dankittipakul & Jocqué, 2004 (Araneae, Zodariidae), with a first description of the male

**DOI:** 10.3897/BDJ.13.e147095

**Published:** 2025-03-26

**Authors:** Lijun Gong, Yang Zhong

**Affiliations:** 1 School of Nuclear Technology and Chemistry & Biology, Hubei University of Science and Technology, Xianning, China School of Nuclear Technology and Chemistry & Biology, Hubei University of Science and Technology Xianning China

**Keywords:** new record, morphology, ant-eating spiders, taxonomy, Thailand

## Abstract

**Background:**

*Euryeidonconsideratum* Dankittipakul & Jocqué, 2004 was described, based on a single female from Doi Luang National Park, Thailand and no additional specimens have been recorded since.

**New information:**

*Euryeidonconsideratum* is re-described and illustrated, based on new material from Xishuangbanna, China. The male is described and illustrated for the first time. In addition, this paper further illustrates the female and provides a supplementary description, confirming that the median plate of the epigyne is absent in this species, rather than broken off as suggested in the original publication. The distribution map of this species is given.

## Introduction

*Euryeidon* Dankittipakul & Jocqué, 2004 is a relatively small genus, with only seven species described so far (WSC 2025). Except for *E.dian* Lu & Li, 2023 from China, all other *Euryeidon* species were described by Dankittipakul and Jocqué (2004) and distributed exclusively in Thailand; amongst them, four were known from both sexes (*E.anthonyi*, *E.dian*, *E.monticola* and *E.musicum*), two from females only (*E.consideratum* and *E.schwendingeri*) and one from male only (*E.sonthichaiae*) (Lu et al. 2023, WSC 2025).

*Euryeidonconsideratum* was first described, based on only one female specimen from Doi Luang National Park, Thailand (Dankittipakul and Jocqué 2004). The examination of spiders collected from the Xishuangbanna rainforest revealed several zodariid individuals of both sexes co-occurring in the same location. Based on the somatic and genital characters, we identified the female as *E.consideratum*. The habitus pattern and leg spination of the male specimen are similar to the female and palpal structures conform to the genus *Euryeidon*. As a result, we matched the females and male together as *E.consideratum*. The goal of this work is to (re-)describe this poorly-known species and its unknown male.

## Materials and methods

Specimens were collected by hand searching on leaf litter. All the examined materials are deposited in The School of Nuclear Technology and Chemistry & Biology, Hubei University of Science and Technology (**HUST**), in Xianning, Hubei, China.

Specimens were examined with an Olympus SZX7 stereomicroscope; details were studied with an Olympus BX41 compound microscope. Male palps and epigynes were examined and illustrated after being dissected. Epigynes were removed and cleared in warm lactic acid before illustration. Photos were made with a Cannon EOS70D digital camera mounted on an Olympus CX41 compound microscope. The digital images were taken and assembled using Helifocus 3.10.3. software package (Khmelik et al. 2005).

The distribution map was generated with ArcGis ver.10.5 (ESRI Inc 2002). Due to the lack of locality coordinates in the previous publication, locality coordinates for *E.consideratum* in Doi Luang National Park, Thailand was originated from Google Earth (see Dankittipakul and Jocqué (2004)).

All measurements were obtained using an Olympus SZX7 stereomicroscope and given in millimetres. Eye diameters are taken at widest point. The total body length does not include chelicerae or spinnerets length. Leg lengths are given as total length (femur, patella, tibia, metatarsus, tarsus). The terminology used in text and figure legends follows Dankittipakul and Jocqué (2004) and Lu et al. (2023).

Abbreviations used in the text and figures are as follows: **A** atrium; **ALE** anterior lateral eyes; **AME** anterior median eyes; **AME–AME** distance between AMEs; **AME–ALE** distance between AME and ALE; **C** conductor; **CB** cymbial bulge; **CF** cymbial fold; **CO** copulatory opening; **Cy** cymbium; **DB** dorsal bugle of cymbium; **dRTA** dorsal branch of RTA; **E** embolus; **EB** embolic base; **ET** embolic tip; **Fe** femora; **LB** lateral border; **MOQ** median ocular quadrangle; **MOQA** MOQ anterior width; **MOQL** length of MOQ; **MOQP** MOQ posterior width; **Mt** metatarsi; **Pa** patellae; **PCE** prolateral cymbial extension; **PLE** posterior lateral eyes; **PME** posterior median eyes; **PME–PME** distance between PMEs; **PME–PLE** distance between PME and PLE; **RCE** retrolateral cymbial extension; **RTA** retrolateral tibial apophysis; **Sp** spermatheca; **TA** tegular apophysis; **TB** transverse band; **Ti** tibiae; **vRTA** ventral branch of RTA.

## Taxon treatments

### Euryeidon consideratum

Dankittipakul & Jocqué, 2004

629C61D7-1B95-549B-90CD-DB34060AE4EA

*Euryeidonconsideratum* Dankittipakul & Jocqué, in Dankittipakul & Jocqué, (2004: 764), figs. 38-40 (description of female).

#### Materials

- **Type status:** Other material. **Occurrence:** recordedBy: Yang Zhong; individualID: HUST-2018-ZOEC001 to 005; individualCount: 5; sex: 2 male, 3 females; lifeStage: adult; occurrenceID: 745CF081-62FE-5B5E-AD30-4C04075C22BC; **Taxon:** scientificName: *Euryeidonconsideratum*; order: Araneae; family: Zodariidae; genus: Euryeidon; specificEpithet: *consideratum*; taxonRank: species; scientificNameAuthorship: Dankittipakul & Jocqué; taxonomicStatus: accepted; **Location:** continent: Asia; country: China; countryCode: CHN; stateProvince: Yunnan; municipality: Jinghong; locality: Menglun Town, Secondary tropical montane evergreen broad-leaved forest; verbatimElevation: 876 m; verbatimCoordinates: 21.91355°N, 101.210567°E; decimalLatitude: 21.91355; decimalLongitude: 101.210567; georeferenceProtocol: label; **Identification:** identifiedBy: Yang Zhong; dateIdentified: 15-10-2024; **Event:** samplingProtocol: hand searching; samplingEffort: 10 km by foot; eventDate: 6/15/2018; **Record Level:** language: en; basisOfRecord: PreservedSpecimen
- **Type status:** Other material. **Occurrence:** recordedBy: Yang Zhong; individualID: HUST-2018-ZOEC006 to 009; individualCount: 4; sex: 2 male, 2 females; lifeStage: adult; occurrenceID: 1433FEC7-8E83-5C0F-8455-B7145EB8FA14; **Taxon:** scientificName: *Euryeidonconsideratum*; order: Araneae; family: Zodariidae; genus: Euryeidon; specificEpithet: *consideratum*; taxonRank: species; scientificNameAuthorship: Dankittipakul & Jocqué; taxonomicStatus: accepted; **Location:** continent: Asia; country: China; countryCode: CHN; stateProvince: Yunnan; municipality: Jinghong; locality: Menglun Town, Paramichelia baillonii plantation; verbatimElevation: 608 m; verbatimCoordinates: 21.903333°N, 101.28205°E; decimalLatitude: 21.903333; decimalLongitude: 101.28205; georeferenceProtocol: label; **Identification:** identifiedBy: Yang Zhong; dateIdentified: 15-10-2024; **Event:** samplingProtocol: hand searching; samplingEffort: 10 km by foot; eventDate: 6/16/2018; **Record Level:** language: en; basisOfRecord: PreservedSpecimen

#### Description

**Figure 1.**
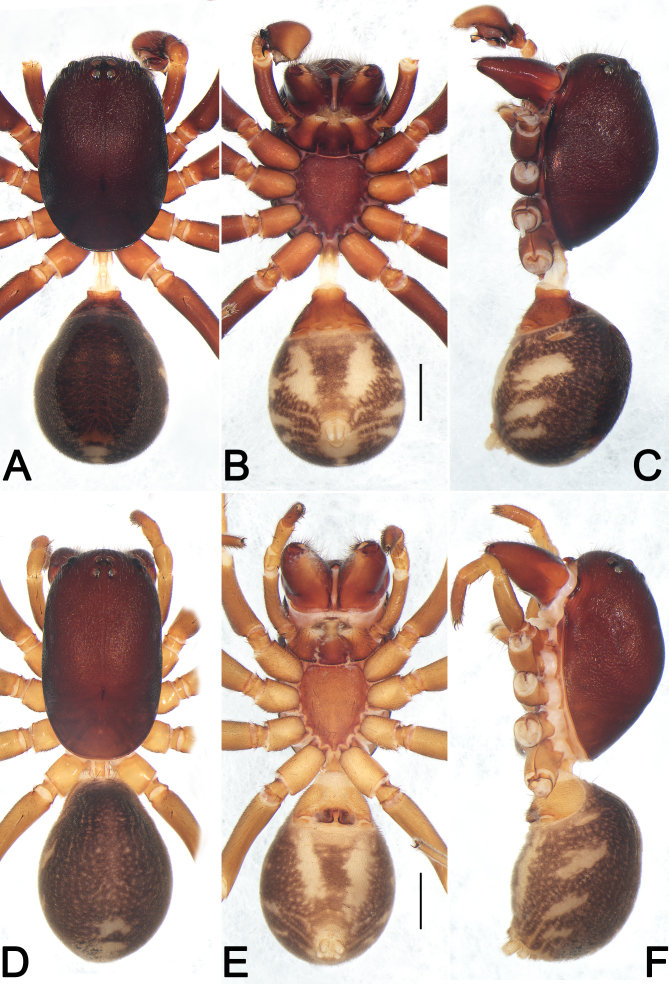
Habitus of *Euryeidonconsideratum*, male (HUST-2018-ZOEC001, **A–C**) and female (HUST-2018-ZOEC002, **D–F**) **A, D** Dorsal view; **B, E** Ventral view; **C, F** Lateral view. Scale bars: 1 mm (equal for **A–C**, equal for **D–F**).

**Figure 2.**
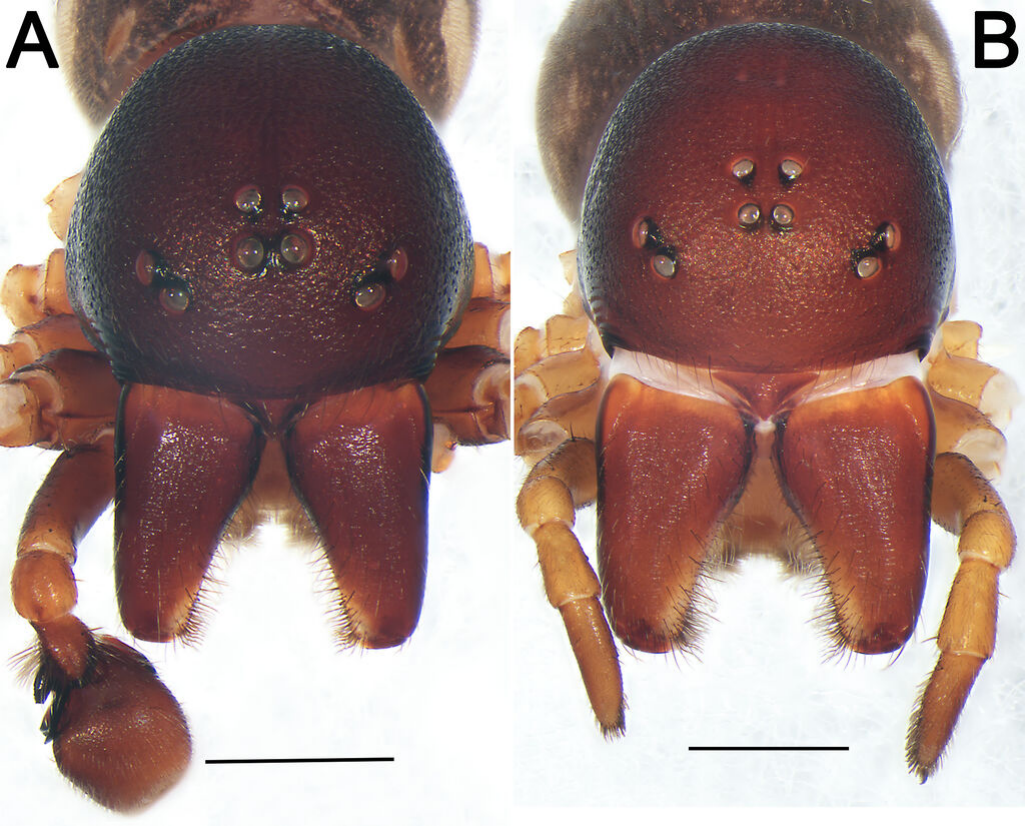
Frontal views of prosoma of *Euryeidonconsideratum*. **A** Male (HUST-2018-ZOEC001); **B** Female (HUST-2018-ZOEC002). Scale bars: 1 mm.

***Male*** (HUST-2018-ZOEC001) (Fig. 1A–C and Fig. 2A). Total length 6.87; prosoma 3.33 long, 2.21 wide, 2.05 high; opisthosoma 2.87 long, 2.30 wide. Eye sizes and interdistances: AME 0.16, ALE 0.18, PME 0.14, PME 0.16, AME–AME 0.08, AME–ALE 0.33, PME–PME 0.11, PME–PLE 0.37. MOQL 0.44; MOQA 0.38, MOQP 0.39. Leg formula 4123; measurements: I 6.75 (1.86, 0.78, 1.50, 1.35, 1.26), II 5.93 (1.65, 0.80, 1.25, 1.20, 1.03), III 5.82 (1.64, 0.77, 1.11, 1.43, 0.87), IV 7.77 (2.07, 0.72, 1.59, 2.11, 1.28). Spination: Fe Ⅰ d11 Ⅱ d11 Ⅲ d111 Ⅳ d111; Pa Ⅲ p1 Ⅳ p1; Ti Ⅰ v222 Ⅱ v222 Ⅲ p111 d111 r111 v2112 Ⅳ p111 d111 r111 v2112; Mt Ⅰ v222 Ⅱ v222 Ⅲ p112 r112 v122 Ⅳ p112 r112 v122.

**Shape and colouration pattern** (Fig. 1A–C and Fig. 2A). Carapace oblong, surface rough, covered with short fine hairs in cephalic area; deep brown, without pattern; in profile strongly domed, highest just in front of longitudinal fovea; cervical groove and radial grooves indistinct, fovea represented by a distinct, longitudinal pit. Chelicerae red wine-coloured, with two promarginal teeth and without retromarginal teeth. Labium triangular, reddish-brown, apically with narrow membranous area and anteromedian scopula, basal and lateral margins distinctly darker. Chilum single sclerite, triangular, elevated and smooth. Endites nearly trapeziform, slightly curved, reddish-orange, proximallly distinctly darker, distally distinctly lighter. Sternum 1.37 long, 1.18 wide, coloured as chelicerae, shield-shaped, pre- and intercoxal triangular sclerites present, anterior margin slightly procurved, with two small indentations at level of labium corner, posterior margin protruding. Legs yellowish-brown, but dark brown on femora. Pedicel cylindrical, weakly sclerotised, relatively short, yellow-brown. Opisthosoma round; dorsum of opisthosoma dark brown, with distinct, oval scutum nearly covering the entire length of the abdomen; laterally with three diagonal, flesh-coloured streaks; venter with two diagonal, flesh-coloured wide streaks. Spinnerets yellowish.

**Figure 3.**
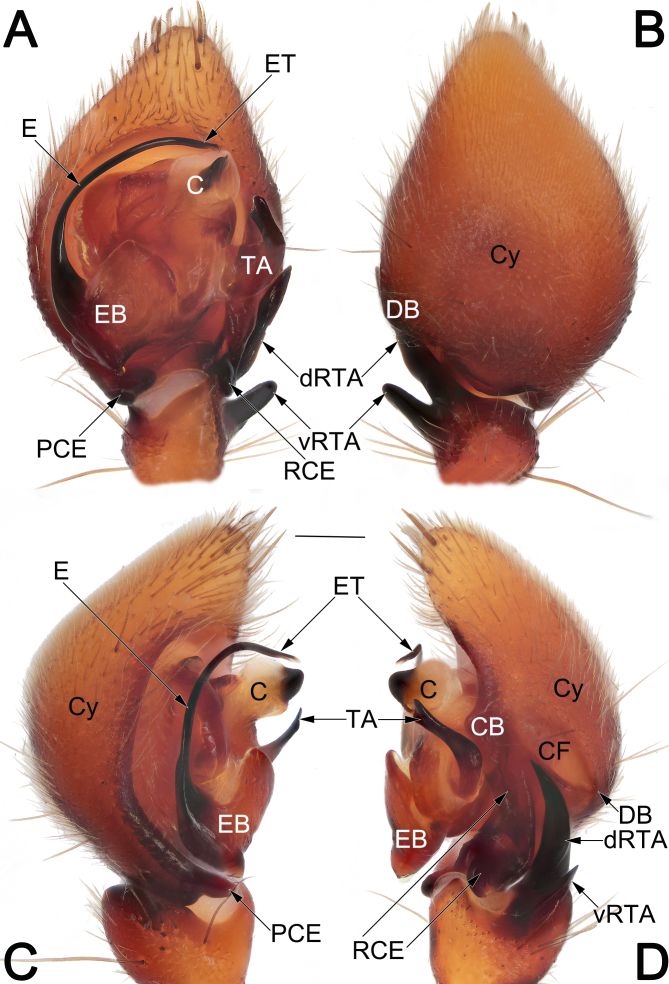
Male palp of *Euryeidonconsideratum* (HUST-2018-ZOEC001). **A** Ventral view; **B** Dorsal view; **C** Prolateral view; **D** Retrolateral view. Scale bar: 0.2 mm (equal for **A–D**). Abbreviations: C, conductor; CB, cymbial bulge; CF, cymbial fold; Cy, cymbium; DB, dorsal bugle of cymbium; dRTA, dorsal branch of RTA; E, embolus; EB, embolic base; ET, embolic tip; PCE, prolateral cymbial extension; RCE, retrolateral cymbial extension; TA, tegular apophysis; vRTA, ventral branch of RTA.

**Figure 4.**
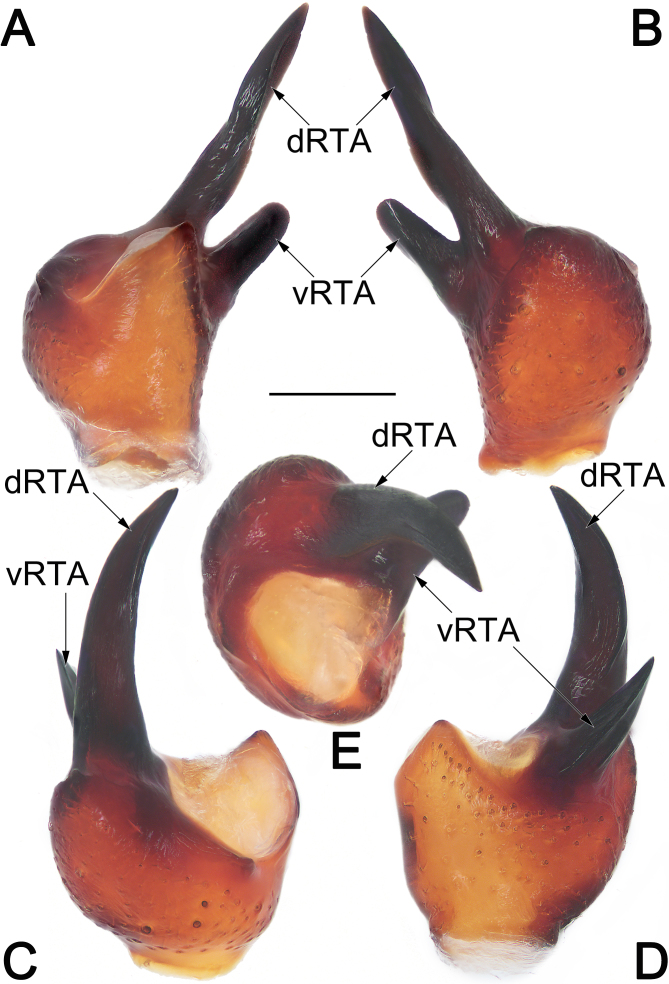
Male palpal tibia of *Euryeidonconsideratum* (HUST-2018-ZOEC001). **A** Ventral view; **B** Dorsal view; **C** Prolateral view; **D** Retrolateral view; **E** Anterior view. Scale bar: 0.2 mm (equal for **A–E**). Abbreviations: dRTA, dorsal branch of RTA; vRTA, ventral branch of RTA.

**Palp** (Fig. 3A–D and Fig. 4A–E). Tibia short, cup-shaped, ca. 1/3 cymbium length, with retrolateral apophysis (RTA) arising retrodistally; RTA deeply bifid, with ventral branch (vRTA) and dorsal branch (dRTA), both vRTA and dRTA being heavily sclerotised, claw-shaped, slightly curved and tapering, apex sharp; dRTA distinctly long, longer than tibia, ca. 2/5 cymbium length, extending to cymbial fold, vRTA relatively shorter, ca. 1/2 dRTA length. Cymbium (Cy) drop-shaped, approximately 1.45 times as long as wide, retrolateral margin with distinct bulge (CB), with pro- (PCE) and retro-basal (RCE) extensions, respectively; PCE distinctly smaller, digitiform, ca. 1/10 cymbium length; RCE as long ridge running along retro-basal margin of cy, ca. 2/5 cy length; cymbial fold (CF) approximately 1/3 length of cymbium (Cy). Tegular apophysis (TA) spatula-like, approximately 1/3 length of tegulum; proximally paler, slightly thicker and curved; medially slender; distally heavily sclerotised and widened, with a flattened apex. Conductor (C) roughly cylindrical; proximal part partly membranous; apical part sclerotised, as a subtriangular flange, apex blunt. Embolus (E) filiform, arising pro-basally at approximately 7–8 o’clock position, curving clockwise along the tegular margin, ending at ca. 1 o’clock position; embolus base (EB) basally with protruding conical extension.

***Female*** (Fig. 1D–E and Fig. 2B). Total length 7.45; prosoma 4.73 long, 2.95 wide; opisthosoma 4.21 long, 3.30 wide, 1.89 high. Eye sizes and interdistances: AME 0.13, ALE 0.15, PME 0.16, PME 0.14, AME–AME 0.07, AME–ALE 0.49, PME–PME 0.16, PME–PLE 0.62. MOQL 0.47; MOQA 0.34, MOQP 0.46. Sternum 1.50 long, 1.25 wide. Leg formula 4123; measurements: I 7.14 (1.97, 0.82, 1.58, 1.40, 1.37), II 6.51 (1.81, 0.9, 1.32, 1.35, 1.13), III 6.30 (1.69, 0.91, 1.12 1.59, 0.99), IV 8.50 (2.23, 0.88, 1.71, 2.3, 1.38). Spination: femora Ⅰ d11 Ⅱ d11 Ⅲ d111 Ⅳ d111; patellae Ⅲ p1 Ⅳ p1; tibiae Ⅰ v222 Ⅱ v1112 Ⅲ p11 d112 r11 v212 Ⅳ p11 d112 r11 v212; metatarsi Ⅰ v222 Ⅱ v222 Ⅲ p111 r111 v112 Ⅳ p111 r111 v112.

**Pattern and colouration** (Fig. 1D–E and Fig. 2B). As in males, but body slightly paler (see Dankittipakul and Jocqué (2004) for additional details).

**Figure 5.**
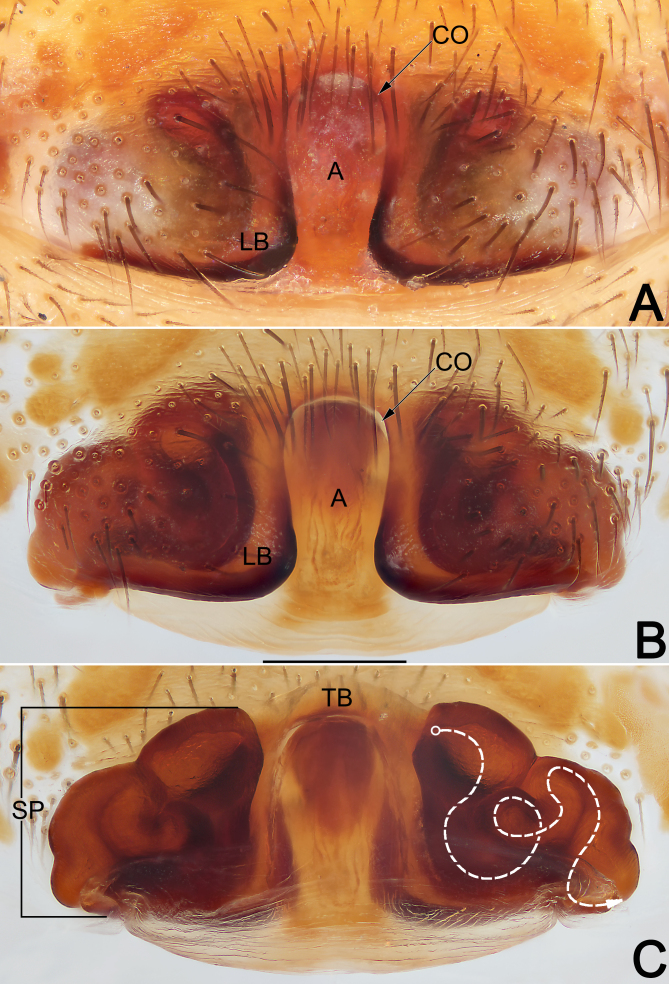
Epigyne of the female of *Euryeidonconsideratum* (HUST-2018-ZOEC002). **A** Epigyne, intact, ventral view; **B** Epigyne, cleared, ventral view; **C** Vulva, cleared, dorsal view. Scale bar: 0.2 mm (equal for **A–C**). Abbreviations: A, atrium; CO, copulatory opening; LB, lateral border; Sp, spermatheca (dashed line showing schematic course of spermatheca, dorsal); TB, transverse band.

**Epigyne** (Fig. 5A–C). Epigynal area sclerotised, ca. twice wider than long. Atrium (A) elongate arch-shaped, ca. 1/5–1/6 epigynal width and 9/10 epigynal length, slightly widened anteriorly; median plate absent. Copulatory openings (CO) indistinct, located anterolaterally under atrial arch. Lateral border (LB) subtriangular, nearly as long as wide, terminally blunt about 80 degrees. Spermathecae (SP) tubular, strongly convoluted, stacked in piles on each side, with thin fertilisation ducts terminally. Anterior transverse (TB) hyaline, arch-shaped.

#### Diagnosis

Males of *E.consideratum* resemble those of *E.sonthichaiae* in the general shape of the male palp. The palps of the two species share the absence of dorsal tibial apophysis and by the deeply divided retrolateral tibial apophysis (RTA) with dorsal branch (vRTA) distinctly longer than ventral branch (vRTA) (Fig. 3A–D, Fig. 4A–E; Dankittipakul and Jocqué (2004): fig. 36) (vs. dorsal tibial apophysis present, RTA not divided, such as in *E.anthonyi*, *E.dian*, *E.monticola* or RTA bifurcated, but both branches with similar length, such as in *E.musicum*; Dankittipakul and Jocqué (2004): figs. 14, 23 and 31; Lu et al. (2023): figs. 2B and C). Males of *E.consideratum* can be recognised from *E.sonthichaiae* by: (1) dRTA longer than palpal tibia, ca. 2/5 cymbium length, extending to cymbial fold (vs. shorter than palpal tibia, ca. 1/10 cymbium length) (cf. Fig. 3A, B, D, Fig. 4A–D and Dankittipakul and Jocqué (2004): fig. 36); (2) tegular apophysis (TA) distinctly curved, with widened apex pointing ventrally and not extending beyond the retrolateral margin of cymbium in ventral view (vs. moderately curved, with tapered apex pointing ventro-retrolaterally and extending distinctly beyond retrolateral margin of cymbium in ventral view) (cf. Fig. 3A and Dankittipakul and Jocqué (2004): fig. 35). Females of *E.consideratum* can be easily distinguished from those of all other congers, with the exception of *E.dian*, by their epigynes without median plate (Fig. 5A, B and Lu et al. (2023): fig. 1E) (vs. median plate with variable shapes, but present, such as in *E.anthonyi*, *E.monticola*, *E.musicum* and *E.schwendingeri*; *Dankittipakul and Jocqué (2004)*: figs. 15, 26, 33 and 41), but can be separated from the latter by the elongated arch-shaped atrium (vs. cordiform) (cf. Fig. 5A, B and Lu et al. (2023): fig. 1E), the lateral borders not touching each other (vs. touching) (cf. Fig. 5A, B and Lu et al. (2023): fig. 1E) and by the tubular spermathecae (vs. round) (cf. Fig. 5C and Lu et al. (2023): fig. 1F).

#### Distribution

**Figure 6.**
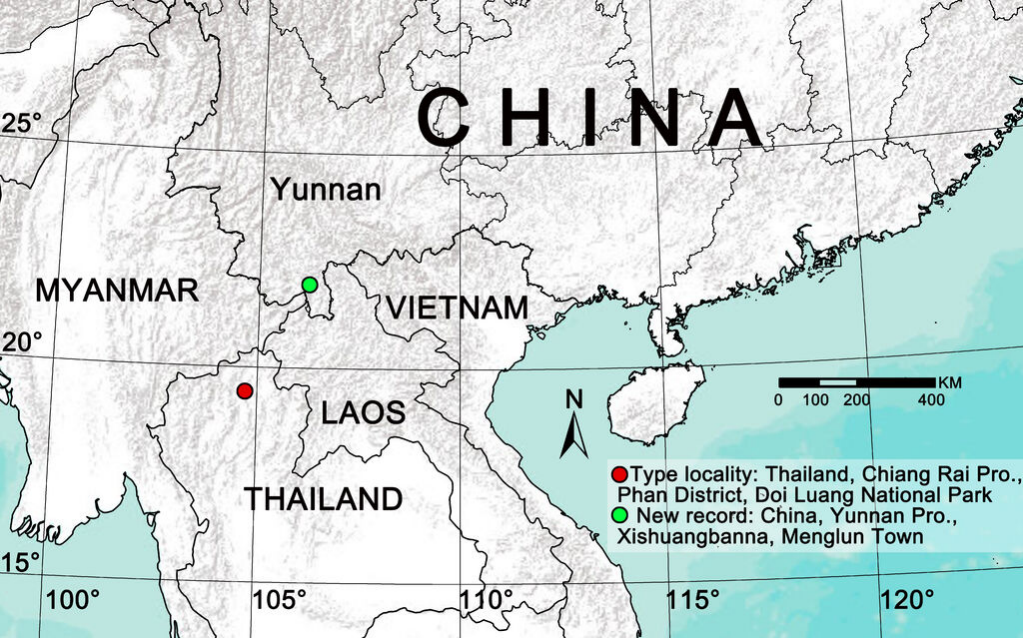
Distribution records of *Euryeidonconsideratum*.

China (Yunnan, new record), Thailand. The new collections extend the known range of this species by ~ 320 km to the northwest (Xishuangbanna) from the type locality (Doi Luang National Park) (Fig. 6).

#### Biology

The specimens were collected in leaf litter.

#### Notes

Dankittipakul and Jocqué (2004) noted 'females have a simple epigyne with a median plate of variable shape' as one of the diagnostic characteristics of the genus *Euryeidon*. In their original paper, all species described, except for *E.consideratum*, exhibited this feature in females (Dankittipakul and Jocqué (2004): figs. 15, 26, 33 and 41). To address this discrepancy, Dankittipakul and Jocqué (2004) suggested that the median plate of *E.consideratum* was 'broken off' rather than absent, based on observations of the holotype female. In the present study, we examined five female specimens of *E.consideratum*, all of which, like the holotype, lacked a median plate. We propose that this structure is likely absent inherently rather than broken off in these specimens. Additionally, the recently-described species *E.dian* also lacks a median plate in its epigyne (Lu et al. (2023): fig. 1E), demonstrating that the median plate is not a synapomorphic feature of the genus.

## Supplementary Material

XML Treatment for Euryeidon consideratum
